# (*E*)-4-Meth­oxy-*N*′-(3,4,5-trihy­droxy­benzyl­idene)benzohydrazide methanol monosolvate

**DOI:** 10.1107/S1600536812036550

**Published:** 2012-09-05

**Authors:** Muhammad Taha, Humera Naz, Aqilah Abd Rahman, Nor Hadiani Ismail, Sammer Yousuf

**Affiliations:** a Atta-ur-Rahman Research Institute for Natural Products Discovery (RiND), Universiti Tecknologi MARA, Puncak Alam, 42300 Selangor, Malaysia; b Faculty of Pharmacy, Universiti Tecknologi MARA, Puncak Alam, 42300 Selangor, Malaysia; c H.E.J. Research Institute of Chemistry, International Center for Chemical and Biological Sciences, University of Karachi, Karachi 75270, Pakistan

## Abstract

The title compound, C_15_H_14_N_2_O_5_·CH_3_OH, displays an *E* conformation about the azomethine double bond [C=N = 1.277 (2) Å] and the benzene rings are inclined to one another by 18.28 (9)°. An intra­molecular O—H⋯O hydrogen bond occurs between the *para*-OH group and one of the *meta*-O atoms of the 3,4,5-trihy­droxy­benzyl­idene group. In the crystal, the components are linked into a three dimensional network by O—H⋯O, O—H⋯N and C—H⋯O hydrogen bonds.

## Related literature

For the biological activity of benzohydrazides see: Khan *et al.* (2012 ▶). For a related structure, see: Bao & Wei (2008 ▶).
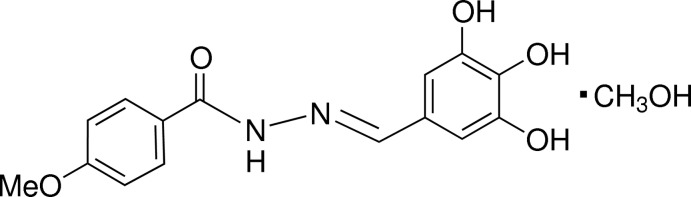


## Experimental

### 

#### Crystal data

C_15_H_14_N_2_O_5_·CH_4_O
*M* *_r_* = 334.32
Monoclinic, 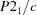

*a* = 11.1846 (7) Å
*b* = 11.1909 (7) Å
*c* = 13.1806 (8) Å
β = 110.368 (1)°
*V* = 1546.61 (17) Å^3^
*Z* = 4
Mo *K*α radiation
μ = 0.11 mm^−1^
*T* = 298 K
0.20 × 0.14 × 0.08 mm

#### Data collection

Bruker SMART APEX CCD diffractometer
Absorption correction: multi-scan (*SADABS*; Bruker, 2000 ▶) *T*_min_ = 0.978, *T*_max_ = 0.991
8994 measured reflections
2787 independent reflections
2230 reflections with *I* > 2σ(*I*)
*R*_int_ = 0.026

#### Refinement

*R*[*F* ^2^ > 2σ(*F* ^2^)] = 0.042
*wR*(*F* ^2^) = 0.114
*S* = 1.03
2787 reflections
239 parameters
H atoms treated by a mixture of independent and constrained refinement
Δρ_max_ = 0.22 e Å^−3^
Δρ_min_ = −0.23 e Å^−3^

### 

Data collection: *SMART* (Bruker, 2000 ▶); cell refinement: *SAINT* (Bruker, 2000 ▶); data reduction: *SAINT*; program(s) used to solve structure: *SHELXS97* (Sheldrick, 2008 ▶); program(s) used to refine structure: *SHELXL97* (Sheldrick, 2008 ▶); molecular graphics: *SHELXTL* (Sheldrick, 2008 ▶); software used to prepare material for publication: *SHELXTL*, *PARST* (Nardelli, 1995 ▶) and *PLATON* (Spek, 2009 ▶).

## Supplementary Material

Crystal structure: contains datablock(s) global, I. DOI: 10.1107/S1600536812036550/hb6938sup1.cif

Structure factors: contains datablock(s) I. DOI: 10.1107/S1600536812036550/hb6938Isup2.hkl

Supplementary material file. DOI: 10.1107/S1600536812036550/hb6938Isup3.cml

Additional supplementary materials:  crystallographic information; 3D view; checkCIF report

## Figures and Tables

**Table 1:** Hydrogen-bond geometry (Å, °)

*D*—H⋯*A*	*D*—H	H⋯*A*	*D*⋯*A*	*D*—H⋯*A*
O1—H1*A*⋯O4^i^	0.77 (3)	2.52 (2)	3.165 (2)	142 (2)
O1—H1*A*⋯N1^i^	0.77 (2)	2.36 (3)	3.040 (2)	147 (2)
O2—H2*A*⋯O1	0.79 (2)	2.20 (2)	2.643 (2)	117 (2)
O2—H2*A*⋯O4^ii^	0.79 (2)	2.22 (2)	2.877 (2)	142 (2)
O3—H3*A*⋯O4^iii^	0.86 (2)	1.93 (2)	2.782 (2)	170 (2)
O6—H6*A*⋯O2^iv^	0.91 (3)	1.87 (3)	2.771 (2)	171 (2)
N2—H2*B*⋯O6^v^	0.80 (2)	2.12 (2)	2.893 (2)	162 (2)
C7—H7*A*⋯O6^v^	0.93	2.58	3.297 (3)	134
C14—H14*A*⋯O6^v^	0.93	2.37	3.259 (3)	159
C15—H15*A*⋯O5^vi^	0.96	2.55	3.224 (3)	127

Symmetry codes: (i) 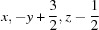; (ii) 
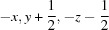
; (iii) 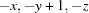; (iv) 
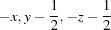
; (v) 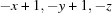; (vi) 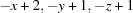.

**Fig. 1.**
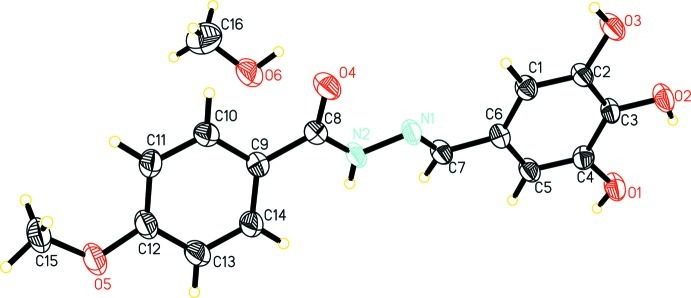
The molecular structure of (I) with displacement ellipsoids drawn at 30% probability level.

**Fig. 2.**
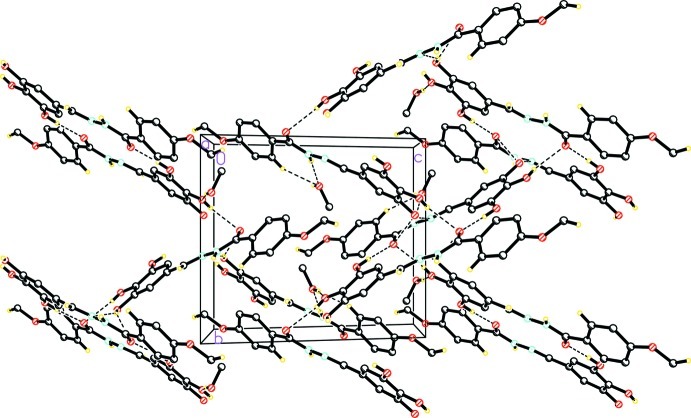
The crystal packing of the title compound I. Only hydrogen atoms involved in hydrogen bonding are shown.

## supplementary crystallographic information

### Comment

The title compound is a phenyl hydrazine derivative synthesized as a part of our ongoing resaerch to study different biological activities of this medicinally important class of organic compounds (Khan *et al.* 2012) and establish an structure activity relationship. The structure of title compound (Fig. 1) is similar to that of the previously published *N*'-(4-Hydroxybenzylidene)-4-methoxybenzohydrazide (Bao *et al.*, 2008) with the difference that 4-Hydroxy benzne ring is replaced by 3,4,5-trihydroxy phenyl ring (C1–C6). The azomethine (C=N,1.277 (2) Å) double bond adopt an *E* configuration (Fig. 1. The benzene rings (C1–C6 and C9–C14) are each almost planner with dihedral angle 18.28 (9)° between them and maximum deviation of -0.015 (2) Å for C11 atom from the root mean square plane of methoxy substituted benzene ring (C9–C14). The bond lengths and angle were found to be similar as in structurally realted compound (Bao *et al.*, 2008). O1—H1A···O4, O1—N1···O4, O2—H2A···O4, O3—H3A···O4, and C15—H15A···O5 hydrogen bonds link the molecules and form a two-dimensional-network, which is further extended to three-dimensional-network due to the intermolecular linkages (N2—H2B···O6, O6—H6A···O2,C7—H7A···O6 and C14—H14A···O6) made by mathanol solvates (symmetry codes as in Table 2 and Fig. 2).

### Experimental

The title compound was synthesized by refluxing in methanol (20 ml) a mixture of 2 mmol of 4-methoxybenzohydrazide (0.332 g), 2 mmol 3,4,5-trihydroxybenzaldehyde monohydrated (0.344 g) and catalytical amount of acetic acid for 3 h. The progress of reaction was monitored by TLC. After completion of reaction, the solvent was evaporated by vacuum to afford crude product which was further recrystallized from methanol solution to obtain colourless plates in 77% yield (0.466 g).

### Refinement

H atoms on Methyl, phenyl and methine were positioned geometrically with C—H = 0.95 Å (CH_3_), and 0.93 Å (CH) and constrained to ride on their parent atoms with *U*_iso_(H)= 1.5*U*_eq_(CH_3_) 1.2*U*_eq_(CH). The H atoms on the nitrogen (N–H= 0.80 (2) Å) and oxygen (O–H= 0.91 (3)–0.77 (3) Å) atoms were located in difference fourier maps and refined isotropically. A rotating group model was applied to the methyl groups.

### Crystal data

**Table:**

C_15_H_14_N_2_O_5_·CH_4_O	*F*(000) = 704
*M**_r_* = 334.32	*D*_x_ = 1.436 Mg m^−^^3^
Monoclinic, *P*2_1_/*c*	Mo *K*α radiation, λ = 0.71073 Å
*a* = 11.1846 (7) Å	Cell parameters from 506 reflections
*b* = 11.1909 (7) Å	θ = 2.9–18.7°
*c* = 13.1806 (8) Å	µ = 0.11 mm^−^^1^
β = 110.368 (1)°	*T* = 298 K
*V* = 1546.61 (17) Å^3^	Plate, colourless
*Z* = 4	0.20 × 0.14 × 0.08 mm

### Data collection

**Table:**

Bruker SMART APEX CCD diffractometer	2787 independent reflections
Radiation source: fine-focus sealed tube	2230 reflections with *I* > 2σ(*I*)
Graphite monochromator	*R*_int_ = 0.026
ω scan	θ_max_ = 25.5°, θ_min_ = 1.9°
Absorption correction: multi-scan (*SADABS*; Bruker, 2000)	*h* = −13→13
*T*_min_ = 0.978, *T*_max_ = 0.991	*k* = −13→13
8994 measured reflections	*l* = −15→14

### Refinement

**Table:**

Refinement on *F*^2^	Primary atom site location: structure-invariant direct methods
Least-squares matrix: full	Secondary atom site location: difference Fourier map
*R*[*F*^2^ > 2σ(*F*^2^)] = 0.042	Hydrogen site location: inferred from neighbouring sites
*wR*(*F*^2^) = 0.114	H atoms treated by a mixture of independent and constrained refinement
*S* = 1.03	*w* = 1/[σ^2^(*F*_o_^2^) + (0.0619*P*)^2^ + 0.3148*P*] where *P* = (*F*_o_^2^ + 2*F*_c_^2^)/3
2787 reflections	(Δ/σ)_max_ < 0.001
239 parameters	Δρ_max_ = 0.22 e Å^−^^3^
0 restraints	Δρ_min_ = −0.23 e Å^−^^3^

### Special details

**Table:**

Geometry. All e.s.d.'s (except the e.s.d. in the dihedral angle between two l.s. planes) are estimated using the full covariance matrix. The cell e.s.d.'s are taken into account individually in the estimation of e.s.d.'s in distances, angles and torsion angles; correlations between e.s.d.'s in cell parameters are only used when they are defined by crystal symmetry. An approximate (isotropic) treatment of cell e.s.d.'s is used for estimating e.s.d.'s involving l.s. planes.
Refinement. Refinement of *F*^2^ against ALL reflections. The weighted *R*-factor *wR* and goodness of fit *S* are based on *F*^2^, conventional *R*-factors *R* are based on *F*, with *F* set to zero for negative *F*^2^. The threshold expression of *F*^2^ > σ(*F*^2^) is used only for calculating *R*-factors(gt) *etc*. and is not relevant to the choice of reflections for refinement. *R*-factors based on *F*^2^ are statistically about twice as large as those based on *F*, and *R*- factors based on ALL data will be even larger.

### Fractional atomic coordinates and isotropic or equivalent isotropic displacement parameters (Å^2^)

**Table:**

	*x*	*y*	*z*	*U*_iso_*/*U*_eq_	
O1	0.01454 (13)	0.88419 (13)	−0.45071 (12)	0.0441 (4)	
O2	−0.21003 (12)	0.80276 (13)	−0.45786 (12)	0.0444 (4)	
O3	−0.23582 (12)	0.64930 (13)	−0.31207 (12)	0.0433 (4)	
O4	0.27016 (11)	0.46606 (12)	0.13725 (10)	0.0392 (4)	
O5	0.84976 (13)	0.48797 (14)	0.44630 (12)	0.0565 (5)	
N1	0.23081 (13)	0.59842 (13)	−0.03975 (12)	0.0316 (4)	
N2	0.35158 (14)	0.57371 (14)	0.03333 (13)	0.0324 (4)	
C1	−0.00733 (16)	0.64529 (15)	−0.21803 (15)	0.0310 (4)	
H1C	−0.0151	0.5926	−0.1661	0.037*	
C2	−0.11475 (15)	0.68460 (15)	−0.30027 (15)	0.0302 (4)	
C3	−0.10337 (15)	0.76480 (15)	−0.37722 (15)	0.0303 (4)	
C4	0.01662 (17)	0.80498 (15)	−0.37121 (15)	0.0309 (4)	
C5	0.12434 (16)	0.76476 (16)	−0.29044 (15)	0.0334 (4)	
H5A	0.2045	0.7907	−0.2875	0.040*	
C6	0.11294 (15)	0.68481 (15)	−0.21292 (14)	0.0297 (4)	
C7	0.22964 (16)	0.64711 (16)	−0.12773 (15)	0.0319 (4)	
H7A	0.3070	0.6591	−0.1379	0.038*	
C8	0.36450 (15)	0.51158 (14)	0.12353 (14)	0.0285 (4)	
C9	0.49511 (16)	0.50096 (15)	0.20356 (14)	0.0298 (4)	
C10	0.52163 (17)	0.41047 (16)	0.28054 (16)	0.0351 (4)	
H10A	0.4584	0.3553	0.2779	0.042*	
C11	0.63947 (17)	0.40068 (17)	0.36061 (16)	0.0383 (5)	
H11A	0.6558	0.3382	0.4101	0.046*	
C12	0.73406 (17)	0.48409 (17)	0.36761 (16)	0.0395 (5)	
C13	0.71055 (18)	0.57266 (18)	0.28968 (17)	0.0449 (5)	
H13A	0.7745	0.6267	0.2919	0.054*	
C14	0.59328 (17)	0.58108 (16)	0.20925 (16)	0.0385 (5)	
H14A	0.5788	0.6411	0.1576	0.046*	
C15	0.8744 (2)	0.4109 (2)	0.53777 (18)	0.0556 (6)	
H15A	0.9566	0.4291	0.5901	0.083*	
H15B	0.8731	0.3292	0.5150	0.083*	
H15C	0.8100	0.4225	0.5696	0.083*	
O6	0.47061 (15)	0.26843 (15)	0.01508 (15)	0.0603 (5)	
C16	0.4983 (3)	0.1595 (2)	0.0630 (2)	0.0762 (8)	
H16A	0.5879	0.1439	0.0822	0.114*	
H16B	0.4507	0.0991	0.0136	0.114*	
H16C	0.4759	0.1583	0.1270	0.114*	
H2B	0.4110 (19)	0.6045 (17)	0.0225 (16)	0.034 (5)*	
H2A	−0.190 (2)	0.846 (2)	−0.496 (2)	0.058 (8)*	
H1A	0.083 (2)	0.897 (2)	−0.448 (2)	0.057 (8)*	
H3A	−0.238 (2)	0.609 (2)	−0.257 (2)	0.059 (7)*	
H6A	0.384 (3)	0.274 (3)	−0.010 (2)	0.093 (10)*	

### Atomic displacement parameters (Å^2^)

**Table:**

	*U*^11^	*U*^22^	*U*^33^	*U*^12^	*U*^13^	*U*^23^
O1	0.0267 (7)	0.0576 (9)	0.0482 (10)	0.0040 (6)	0.0133 (7)	0.0264 (7)
O2	0.0245 (7)	0.0606 (9)	0.0436 (9)	0.0040 (6)	0.0062 (6)	0.0261 (7)
O3	0.0233 (7)	0.0627 (9)	0.0404 (9)	−0.0039 (6)	0.0067 (6)	0.0198 (7)
O4	0.0273 (7)	0.0557 (8)	0.0321 (8)	−0.0078 (6)	0.0073 (6)	0.0068 (6)
O5	0.0318 (7)	0.0713 (10)	0.0498 (10)	−0.0093 (7)	−0.0066 (7)	0.0251 (8)
N1	0.0213 (7)	0.0384 (8)	0.0298 (9)	0.0029 (6)	0.0024 (7)	0.0042 (7)
N2	0.0179 (7)	0.0440 (9)	0.0319 (9)	−0.0009 (6)	0.0045 (7)	0.0084 (7)
C1	0.0292 (9)	0.0379 (9)	0.0239 (10)	0.0006 (7)	0.0066 (8)	0.0051 (7)
C2	0.0221 (8)	0.0372 (9)	0.0305 (11)	−0.0005 (7)	0.0082 (8)	0.0019 (8)
C3	0.0226 (8)	0.0376 (9)	0.0283 (10)	0.0052 (7)	0.0059 (8)	0.0058 (8)
C4	0.0301 (9)	0.0340 (9)	0.0307 (10)	0.0025 (7)	0.0131 (8)	0.0072 (8)
C5	0.0213 (8)	0.0413 (10)	0.0367 (11)	0.0002 (7)	0.0090 (8)	0.0054 (8)
C6	0.0245 (9)	0.0354 (9)	0.0266 (10)	0.0026 (7)	0.0054 (8)	0.0005 (7)
C7	0.0231 (9)	0.0403 (9)	0.0310 (11)	0.0000 (7)	0.0077 (8)	0.0021 (8)
C8	0.0257 (9)	0.0309 (8)	0.0281 (10)	−0.0005 (7)	0.0084 (8)	−0.0019 (7)
C9	0.0269 (9)	0.0331 (9)	0.0269 (10)	0.0007 (7)	0.0064 (8)	0.0004 (7)
C10	0.0290 (9)	0.0371 (9)	0.0372 (12)	−0.0044 (7)	0.0091 (9)	0.0045 (8)
C11	0.0340 (10)	0.0402 (10)	0.0365 (12)	0.0021 (8)	0.0069 (9)	0.0127 (8)
C12	0.0264 (9)	0.0493 (11)	0.0361 (12)	0.0001 (8)	0.0024 (9)	0.0078 (9)
C13	0.0303 (10)	0.0509 (11)	0.0460 (13)	−0.0109 (8)	0.0037 (9)	0.0118 (10)
C14	0.0338 (10)	0.0419 (10)	0.0340 (12)	−0.0040 (8)	0.0046 (9)	0.0112 (8)
C15	0.0387 (12)	0.0720 (15)	0.0424 (14)	−0.0027 (10)	−0.0030 (10)	0.0214 (11)
O6	0.0305 (8)	0.0738 (11)	0.0694 (12)	−0.0075 (7)	0.0084 (8)	0.0217 (9)
C16	0.0721 (18)	0.0712 (17)	0.091 (2)	−0.0080 (14)	0.0349 (17)	0.0196 (16)

### Geometric parameters (Å, º)

**Table:**

O1—C4	1.366 (2)	C6—C7	1.457 (2)
O1—H1A	0.77 (3)	C7—H7A	0.9300
O2—C3	1.360 (2)	C8—C9	1.479 (2)
O2—H2A	0.78 (3)	C9—C10	1.391 (2)
O3—C2	1.366 (2)	C9—C14	1.399 (2)
O3—H3A	0.86 (3)	C10—C11	1.376 (3)
O4—C8	1.240 (2)	C10—H10A	0.9300
O5—C12	1.348 (2)	C11—C12	1.390 (3)
O5—C15	1.429 (2)	C11—H11A	0.9300
N1—C7	1.277 (2)	C12—C13	1.385 (3)
N1—N2	1.3867 (19)	C13—C14	1.373 (3)
N2—C8	1.341 (2)	C13—H13A	0.9300
N2—H2B	0.80 (2)	C14—H14A	0.9300
C1—C2	1.380 (2)	C15—H15A	0.9600
C1—C6	1.395 (2)	C15—H15B	0.9600
C1—H1C	0.9300	C15—H15C	0.9600
C2—C3	1.393 (2)	O6—C16	1.358 (3)
C3—C4	1.391 (2)	O6—H6A	0.91 (3)
C4—C5	1.376 (2)	C16—H16A	0.9600
C5—C6	1.397 (3)	C16—H16B	0.9600
C5—H5A	0.9300	C16—H16C	0.9600
			
C4—O1—H1A	109.2 (18)	C10—C9—C14	117.61 (16)
C3—O2—H2A	108.8 (18)	C10—C9—C8	119.06 (15)
C2—O3—H3A	111.6 (16)	C14—C9—C8	123.27 (16)
C12—O5—C15	118.75 (15)	C11—C10—C9	121.43 (16)
C7—N1—N2	114.55 (14)	C11—C10—H10A	119.3
C8—N2—N1	119.84 (15)	C9—C10—H10A	119.3
C8—N2—H2B	122.6 (14)	C10—C11—C12	120.08 (17)
N1—N2—H2B	117.2 (14)	C10—C11—H11A	120.0
C2—C1—C6	119.86 (16)	C12—C11—H11A	120.0
C2—C1—H1C	120.1	O5—C12—C13	115.53 (16)
C6—C1—H1C	120.1	O5—C12—C11	125.27 (17)
O3—C2—C1	123.67 (16)	C13—C12—C11	119.19 (17)
O3—C2—C3	116.20 (15)	C14—C13—C12	120.36 (17)
C1—C2—C3	120.13 (15)	C14—C13—H13A	119.8
O2—C3—C4	120.65 (16)	C12—C13—H13A	119.8
O2—C3—C2	119.50 (15)	C13—C14—C9	121.22 (17)
C4—C3—C2	119.84 (16)	C13—C14—H14A	119.4
O1—C4—C5	125.59 (16)	C9—C14—H14A	119.4
O1—C4—C3	114.04 (16)	O5—C15—H15A	109.5
C5—C4—C3	120.37 (16)	O5—C15—H15B	109.5
C4—C5—C6	119.81 (16)	H15A—C15—H15B	109.5
C4—C5—H5A	120.1	O5—C15—H15C	109.5
C6—C5—H5A	120.1	H15A—C15—H15C	109.5
C1—C6—C5	119.98 (16)	H15B—C15—H15C	109.5
C1—C6—C7	122.38 (16)	C16—O6—H6A	106.1 (19)
C5—C6—C7	117.64 (15)	O6—C16—H16A	109.5
N1—C7—C6	123.15 (16)	O6—C16—H16B	109.5
N1—C7—H7A	118.4	H16A—C16—H16B	109.5
C6—C7—H7A	118.4	O6—C16—H16C	109.5
O4—C8—N2	120.50 (16)	H16A—C16—H16C	109.5
O4—C8—C9	122.83 (16)	H16B—C16—H16C	109.5
N2—C8—C9	116.67 (15)		
			
C7—N1—N2—C8	−173.13 (16)	C5—C6—C7—N1	162.91 (17)
C6—C1—C2—O3	179.17 (17)	N1—N2—C8—O4	7.0 (3)
C6—C1—C2—C3	−0.9 (3)	N1—N2—C8—C9	−173.02 (15)
O3—C2—C3—O2	−0.1 (3)	O4—C8—C9—C10	19.5 (3)
C1—C2—C3—O2	179.97 (16)	N2—C8—C9—C10	−160.50 (16)
O3—C2—C3—C4	−179.83 (17)	O4—C8—C9—C14	−157.59 (18)
C1—C2—C3—C4	0.2 (3)	N2—C8—C9—C14	22.5 (3)
O2—C3—C4—O1	0.9 (3)	C14—C9—C10—C11	0.9 (3)
C2—C3—C4—O1	−179.34 (16)	C8—C9—C10—C11	−176.26 (17)
O2—C3—C4—C5	−178.95 (17)	C9—C10—C11—C12	1.7 (3)
C2—C3—C4—C5	0.8 (3)	C15—O5—C12—C13	171.0 (2)
O1—C4—C5—C6	179.03 (17)	C15—O5—C12—C11	−8.2 (3)
C3—C4—C5—C6	−1.2 (3)	C10—C11—C12—O5	175.53 (19)
C2—C1—C6—C5	0.5 (3)	C10—C11—C12—C13	−3.6 (3)
C2—C1—C6—C7	179.59 (16)	O5—C12—C13—C14	−176.38 (19)
C4—C5—C6—C1	0.5 (3)	C11—C12—C13—C14	2.8 (3)
C4—C5—C6—C7	−178.63 (16)	C12—C13—C14—C9	−0.2 (3)
N2—N1—C7—C6	−177.28 (15)	C10—C9—C14—C13	−1.7 (3)
C1—C6—C7—N1	−16.2 (3)	C8—C9—C14—C13	175.34 (18)

### Hydrogen-bond geometry (Å, º)

**Table:**

*D*—H···*A*	*D*—H	H···*A*	*D*···*A*	*D*—H···*A*
O1—H1*A*···O4^i^	0.77 (3)	2.52 (2)	3.165 (2)	142 (2)
O1—H1*A*···N1^i^	0.77 (2)	2.36 (3)	3.040 (2)	147 (2)
O2—H2*A*···O1	0.79 (2)	2.20 (2)	2.643 (2)	117 (2)
O2—H2*A*···O4^ii^	0.79 (2)	2.22 (2)	2.877 (2)	142 (2)
O3—H3*A*···O4^iii^	0.86 (2)	1.93 (2)	2.782 (2)	170 (2)
O6—H6*A*···O2^iv^	0.91 (3)	1.87 (3)	2.771 (2)	171 (2)
N2—H2*B*···O6^v^	0.80 (2)	2.12 (2)	2.893 (2)	162 (2)
C7—H7*A*···O6^v^	0.93	2.58	3.297 (3)	134
C14—H14*A*···O6^v^	0.93	2.37	3.259 (3)	159
C15—H15*A*···O5^vi^	0.96	2.55	3.224 (3)	127

Symmetry codes: (i) *x*, −*y*+3/2, *z*−1/2; (ii) −*x*, *y*+1/2, −*z*−1/2; (iii) −*x*, −*y*+1, −*z*; (iv) −*x*, *y*−1/2, −*z*−1/2; (v) −*x*+1, −*y*+1, −*z*; (vi) −*x*+2, −*y*+1, −*z*+1.
